# Atypical Pestivirus and Severe Respiratory Disease in Calves, Europe

**DOI:** 10.3201/eid1811.111298

**Published:** 2012-11

**Authors:** Lihong Liu, Magdalena Larska, Hongyan Xia, Åse Uttenthal, Miroslaw P. Polak, Karl Ståhl, Stefan Alenius, Hu Shan, Hong Yin, Sándor Belák

**Affiliations:** National Veterinary Institute, Uppsala, Sweden (L. Liu, S. Belák);; National Veterinary Research Institute, Pulawy, Poland (M. Larska, M.P. Polak);; Swedish University of Agricultural Sciences, Uppsala (M. Larska, H. Xia, K. Ståhl, S. Alenius, S. Belák);; Technical University of Denmark, Lindholm, Denmark (A. Uttenthal);; Qingdao Agricultural University, Qingdao, People’s Republic of China (H. Shan);; and Lanzhou Veterinary Research Institute, Lanzhou, China (H. Yin)

**Keywords:** atypical pestivirus, atypical bovine pestiviruses, pestivirus, Hobi-like pestivirus, viruses, bovine viral diarrhea virus, disease, calves, Italy

**To the Editor:** The article by Decaro et al. (*1*) described an outbreak of severe respiratory disease in calves in Italy caused by an atypical bovine pestivirus. This report confirms our concern that this group of viruses is probably widespread (*2*) and present on >3 continents. Moreover, it demonstrates that atypical bovine pestiviruses are capable of causing disease in calves in the field and a clinical picture consistent with most bovine viral diarrhea virus (BVDV) infections that occur naturally or experimentally (*3*,*4*).

Some key issues remain unknown. The origin of the bovine pestivirus and the route of introduction into the herd are unclear, although phylogenies demonstrated a close relationship between this virus strain from Italy and atypical bovine pestiviruses from Brazil. Batches of fetal bovine serum from Brazil have repeatedly been found to be contaminated with atypical bovine pestiviruses (*2*,*3*), and there is a risk for contamination of vaccines with these viruses. Animal trade and vaccines should be considered when conducting further investigations into this outbreak.

The evolutionary relationship between the atypical and the recognized pestivirus species (*1*) needs to be clarified. Maximum-likelihood and Bayesian analyses of a concatenated dataset positioned atypical pestiviruses consistently in a clade sister to BVDV-1 and BVDV-2, and the larger clade was sister to pestivirus of giraffes (*5*). The same pattern was observed in complete genome phylogeny (*1*).

The term atypical is not informative, and a new name has been proposed for these bovine pestiviruses (*5*). Outcomes of infections with these viruses are typical, but the viral antigens and phylogenies are unique.
