# Supplementary material for: Atypical Pestivirus and Severe Respiratory Disease in Calves, Europe
Source: Emerg Infect Dis. 2012 Nov;18(11):1917–8. doi: 10.3201/eid1811.111298 (PMC3559163; doi:10.3201/eid1811.111298)
Supplement: Related texts

# Reply: Atypical Pestivirus and Severe Respiratory Disease in Calves, Europe

**DOI:** 10.3201/eid1811.121112

**Authors:** Nicola Decaro, Canio Buonavoglia

**Affiliations:** University of Bari, Bari, Italy

**In Response:** We read with interest the comment by Liu et al. (*1*) to our article reporting overt disease in calves caused by infection with atypical or ‘Hobi’-like pestivirus (*2*). Although we can rule out that vaccination was the source of infection because only killed vaccines were administered on the farm, the most recent findings about this emerging group of viruses deserves further discussion regarding their possible taxonomical, diagnostic, and prophylactic implications.

The association of ‘Hobi’-like pestivirus with clinical disease in cattle was confirmed by reports of experimental and natural infections (*3*,*4*). A cytopathogenic/noncytopathogenic ‘Hobi’-like virus pair was isolated from a diseased heifer, demonstrating that the new group of viruses includes 2 biotypes already known for bovine viral diarrheal viruses 1 (BVDV-1) and 2 (BVDV-2) (*5*).

Although the clinical presentation might mimic that of extant BVDV-1/BVDV-2, distinctive features were also evident in the reported cases (*1**,**4*). At the antigenic and phylogenetic levels, ‘Hobi’-like strains showed distinct differences from the other bovine pestiviruses: they were at least as different from BVDV-1/2 as are border disease virus and classical swine fever virus (*6*; N. Decaro et al., unpub. data). The antigenic and genetic uniqueness of ‘Hobi’-like viruses will likely lead to the definition of a new species within genus *Pestivirus,* but how this species should be designated remains unclear.

Some scientists have tentatively proposed to name the virus bovine viral diarrhea 3, but others believe this nomenclature would be problematic from regulatory and scientific standpoints (J. Ridpath, pers. comm.). Molecular assays standardized for BVDV-1/2 might not be able to detect ‘Hobi’-like strains because of the presence of mismatches in the oligonucleotide binding regions (*7**)*. Prophylactic measures should take into account the circulation of ‘Hobi’-like pestiviruses in cattle herds. Whether commercial BVDV vaccines are effective against the emerging pestivirus is unknown, and requires future in-vivo cross-protection studies.

## References

[1] Liu L, Larska M, Xia H, Uttenthal Å, Polak MP, Ståhl K, Atypical pestivirus and severe respiratory disease in calves, Europe [letter]. Emerg Infect Dis. 2012;18:1917.2309260410.3201/eid1811.111298PMC3559163

[2] Decaro N, Lucente MS, Mari V, Cirone F, Cordioli P, Camero M, Atypical pestivirus and severe respiratory disease in calves, Europe. Emerg Infect Dis. 2011;17:1549–52.10.3201/eid1708.10144721801648PMC3381567

[3] Decaro N, Mari V, Lucente MS, Sciarretta R, Moreno A, Armenise C, Experimental infection of cattle, sheep and pigs with ‘Hobi’-like pestivirus. Vet Microbiol. 2012;155:165–71. 10.1016/j.vetmic.2011.08.03021955447PMC7126764

[4] Decaro N, Lucente MS, Mari V, Sciarretta R, Pinto P, Buonavoglia D, 'Hobi'-like pestivirus in aborted bovine fetuses. J Clin Microbiol. 2012;50:509–12. 10.1128/JCM.05887-1122162547PMC3264192

[5] Decaro N, Mari V, Pinto P, Lucente MS, Sciarretta R, Cirone F, ‘Hobi’-like pestivirus: both biotypes isolated from diseased animal. J Gen Virol. 2012;•••:93.2276431910.1099/vir.0.044552-0

[6] Bauermann FV, Flores EF, Ridpath JF. Antigenic relationships between Bovine viral diarrhea virus 1 and 2 and HoBi virus: possible impacts on diagnosis and control. J Vet Diagn Invest. 2012;24:253–61. 10.1177/104063871143514422379042

[7] Decaro N, Sciarretta R, Lucente MS, Mari V, Amorisco F, Colaianni ML, A nested PCR approach for unambiguous typing of pestiviruses infecting cattle. Mol Cell Probes. 2012c;26:42–6. 10.1016/j.mcp.2011.11.00322119481PMC7127541
